# Functional Deficiency of Interneurons and Negative BOLD fMRI Response

**DOI:** 10.3390/cells12050811

**Published:** 2023-03-06

**Authors:** Daniil P. Aksenov, Limin Li, Natalya A. Serdyukova, David A. Gascoigne, Evan D. Doubovikov, Alexander Drobyshevsky

**Affiliations:** 1 Department of Radiology, NorthShore University HealthSystem, Evanston, IL 60201, USA; 2 Department of Anesthesiology, NorthShore University HealthSystem, Evanston, IL 60201, USA; 3 Pritzker School of Medicine, University of Chicago, Chicago, IL 60637, USA; 4 Department of Biomedical Engineering, Northwestern University, Evanston, IL 60208, USA; 5 Department of Pediatrics, NorthShore University HealthSystem, Evanston, IL 60201, USA

**Keywords:** rabbit, neurovascular coupling, picrotoxin, somatosensory, GABA, excitatory-inhibitory balance

## Abstract

The functional deficiency of the inhibitory system typically appears during development and can progress to psychiatric disorders or epilepsy, depending on its severity, in later years. It is known that interneurons, the major source of GABAergic inhibition in the cerebral cortex, can make direct connections with arterioles and participate in the regulation of vasomotion. The goal of this study was to mimic the functional deficiency of interneurons through the use of localized microinjections of the GABA antagonist, picrotoxin, in such a concentration that it did not elicit epileptiform neuronal activity. First, we recorded the dynamics of resting-state neuronal activity in response to picrotoxin injections in the somatosensory cortex of an awake rabbit; second, we assessed the altered neuronal and hemodynamic responses to whisker stimulation using BOLD fMRI and electrophysiology recordings; third, we evaluated brain tissue oxygen levels before and after picrotoxin injection. Our results showed that neuronal activity typically increased after picrotoxin administration, the BOLD responses to stimulation became negative, and the oxygen response was nearly abolished. Vasoconstriction during the resting baseline was not observed. These results indicate that picrotoxin provoked imbalanced hemodynamics either due to increased neuronal activity, decreased vascular response, or a combination of both.

## 1. Introduction

The functional deficiency of the inhibitory system (FDIS) is a common phenomenon. Often, it appears during development as a result of perinatal brain injury, hypoxia, neonatal anesthesia, etc. [1,2,3,4,5]. If FDIS is severe, in later years, it can develop into epilepsy [6]. Alternatively, if FDIS is mild, it can contribute to psychiatric disorders, including autism and schizophrenia, depending on its location [7,8]. Typically, if FDIS presents during development, it tends to have a very long duration (months and years) [1,9].

It is known that GABAergic interneurons, which are the main source of inhibition in the cerebral cortex, form direct synaptic connections not only with local neurons in the cerebral cortex, but with local blood vessels as well, so that GABAergic terminals can interact directly with the microvascular bed and the somata [10]. Moreover, the function of fast GABA-A receptors on brain microvessels is independent of the total neuronal activity in contrast to GABA-B receptors [11], which additionally suggests that arteriolar GABA-A receptors are modulated by select GABAergic interneurons that make direct contact with these receptors. These connections can control vascular diameter [12,13] and regulate local blood delivery. Interneurons can contribute to both processes: (1) decreasing oxygen consumption by inhibiting neuronal activity and (2) increasing oxygen delivery by participating in vasodilation. This dual effect represents a complex mechanism that can ensure sufficient oxygen delivery to the brain tissue.

The goal of our study was to mimic mild FDIS, which may be present without a clinical manifestation, in the cerebral cortex and evaluate the BOLD fMRI response to normal sensory stimulations. For this purpose, we suppressed local inhibition without provoking any epileptiform electrophysiological events by locally injecting a low concentration of a GABA-antagonist, picrotoxin (PTX). We hypothesized that if interneurons directly participate in the control of the diameter of arterioles, the BOLD fMRI response magnitude would decrease under mild FDIS. Our results show that while neuronal activity typically increased with PTX application, BOLD responses to stimulation became abnormally negative.

## 2. Materials and Methods

### 2.1. Animal Preparation

Female pigmented Dutch-belted rabbits (2–3 kg, 3–6 months of age) were used in these experiments and performed in accordance with the National Institute of Health guidelines, with the approval of NorthShore University Health System’s Institutional Animal Care and Use Committee.

Three groups of animals were used for the experiments. In the first group (N = 3), the dynamics of resting-state neuronal activity after injections of GABA antagonist PTX and GABA-agonist muscimol (MSC) were studied. In the second group, BOLD fMRI and neuronal responses to whisker (N = 5) stimulation were recorded following the injection of PTX. In the third group, brain tissue oxygen (PO_2_) responses were recorded following the injection of PTX (N = 4). The total number of rabbits was 12.

We generally followed our previously described methods to implant the injection cannula and electrodes [14]. Animals were anesthetized with a mixture of ketamine (50 mg/kg) and xylazine (10 mg/kg), and an incision was made in the scalp and the bone was exposed on the top of the skull. A lightweight head restraining device containing four nylon bolts was implanted on the top of the skull. This headbolt was used to secure the radiofrequency (RF) coil and the animal’s head in the same position in order to obtain a reproducible slice positioning among subjects, as described previously [15]. For neuronal recording, the assembly that was implanted consisted of a bundle of four 25 µm diameter gold-silver alloy microwires with formvar insulation (California Fine Wire, Grover Beach, CA, USA) inside a silica tube (Polymicro Technologies, Phoenix, AZ, USA). For PO_2_ recordings, we used gold-plated microwires. The electrode materials were chosen based on our previous evaluation to minimize susceptibility to artifacts in MR images. These electrodes terminated at different levels within a distance of 100 µm and were attached to a permanently implanted custom-made nylon microdrive that permitted vertical adjustments of its position. The microwires were connected to a small 6-pin connector that was embedded in dental acrylic. A 150 µm Ag/AgCl wire was placed between the skull and dura mater to serve as the reference electrode. A 200 µm silica injection cannula was attached to the microdrive. During implantation surgery, the lambda was positioned 1.5 mm below the bregma, and the stereotaxic coordinates were as follows: the anterior-posterior was 2 mm dorsal to bregma, medial-lateral was 6 mm from the midline, and dorsal-ventral was under visual control relative to the surface of the cerebral cortex (the initial position of the electrodes was 0.8 mm from the surface of the brain). Later, the electrodes were advanced to layer IV of the cerebral cortex.

One week after the implantation surgery, each subject (N = 3) in the first group was habituated to the cloth bag and environment for resting state electrophysiology recordings. Additionally, after a one-week recovery period from surgery, each subject from the second group (N = 5) was habituated to the imaging environment for 3–5 days prior to the experiments. The third group (N = 4) was used for the recording of PO_2_ responses. For each MRI or PO_2_ experiment, the rabbits were restrained by means of a cloth sleeve and secured in an acrylic imaging cradle by Velcro straps. The subjects that were used for only electrophysiology recordings were also restrained in the same cloth bag, but the head was not fixed in a cradle.

### 2.2. fMRI Data Collection and Analysis

Imaging was performed using a 9.4 T imaging spectrometer (BioSpec 94/30USR, Bruker, Billerica, MA, USA) operating at a proton frequency of 400 MHz. This system was equipped with an Oxford horizontal magnet and an Acustar actively shielded gradient coil assembly with a clear bore of 15 cm. A flat, circular surface coil (20 mm in diameter) was used for RF transmission and reception. A multi-slice, single-shot gradient echo EPI pulse sequence, with a repetition time (TR) of 2 s and an echo time (TE) of 11 ms, was used to acquire fMRI data. Coronal images in a plane perpendicular to the surface coil were collected from four contiguous slices with a thickness of 1.0 mm, which included the electrode recording site, which used an 80 × 80 matrix size and a 30 mm × 30 mm field of view (FOV), which corresponded to an in-plane resolution of 375 µm × 375 µm. The slices were positioned to include the whisker barrel cortex for vibrissae stimulation experiments. The rabbits were subjected to ten trials in each experiment, during which they were presented with the stimulation paradigm described below. The initial five images for each trial were discarded to ensure that the MR signal reached equilibrium. Prior to the fMRI data acquisition, high-resolution anatomical images (512 × 512 matrix, 48 mm × 48 mm FOV, equivalent to 94 µm × 94 µm in-plane resolution) were also obtained using a multi-slice gradient echo sequence (1.0 mm slice thickness; TR, 1.5 s; TE, 20 ms, NA = 8). The anatomical images were used to identify the optimal slice position of functional images and the location of the electrodes.

The time series fMRI data were registered to the first volume using an affine method that was implemented in the ITK toolkit [16]. Each trial was inspected for residual head movement after registration, and any trials exhibiting movement were excluded from the analysis. The remaining trials were averaged for each experiment. Activated voxels were detected using unsupervised support vector machine (SVM) analysis. Briefly, the mapping process was formulated as an outlier detection problem of one-class SVM, which provided the initial mapping results. These results were further refined by applying a prototype selection and two-class SVM reclassification, as described previously [17]. For better visibility of the changes in BOLD areas, we plotted BOLD activation areas with superimposed correlation coefficients. Activated areas and time courses were then averaged across subjects and expressed as the mean +/− standard error (SEM).

### 2.3. Stimulus Preparation

Whisker stimulation was delivered by an MRI-compatible system that incorporated real-time optical monitoring of the frequency and amplitude to ensure the consistency of the vibration stimulus, as described previously [18]. We have previously evaluated the BOLD response over a range of whisker stimulus frequencies [14]. Based on these results, a whisker stimulation frequency of 50 Hz with +/− 0.3 mm deflection invoked an optimal BOLD response and was used for all the experiments. Typically, three principal whiskers on the left side were stimulated in each experiment, and their selection depended upon the location of the electrode to provide the best response. The stimulation paradigm consisted of a non-stimulus baseline period (25 images = 50 s), a stimulation period (20 images = 40 s), and a post-stimulus period (20 images = 40 s).

### 2.4. Electrophysiological Recording and Microinjections

The electrophysiological signals from the microwires were fed through a miniature preamplifier to a multi-channel differential amplifier system (Neuralynx, Inc, Bozeman, MT, USA). The signals were amplified, band-pass filtered (300 Hz-3 kHz for single units), and digitized (32 kHz/channel) using a Neuralynx data acquisition system. Electrophysiological signals from the neuronal activity were analyzed after the removal of blocks of strong interference signals that are induced by gradient pulses. The gradient pulses refer to the additional magnetic gradient fields that are only applied for a short duration, typically around 30 ms, with rising and falling times of ~100 µs. These blocks were detected by thresholding followed by one-dimensional mathematical morphology [19] and processing based on erosion and dilation functions. To capture the initial neuronal activity without the interfering signals from the gradients, the stimulus onset was delayed for 150 ms from the MR acquisition triggering pulse. Subsequently, unit discrimination was performed offline using threshold detection followed by a cluster analysis of scatter plots of time and amplitude distances between the peak and valley of individual action potential wave shapes. After setting the threshold using CscSpikeExtractor, the data were converted into a SpikeSort 3D datafile. The criteria for the threshold was 3×, an estimate of the standard deviation of the background noise [20]. Spike sorting was performed automatically using KlustaKwik, which is incorporated into SpikeSort 3D. KlustaKwik analysis was followed by manual adjustments of the clusters. We evaluated the interspike interval to ensure that it was no less than 1 ms. We attempted to sort single units into pyramidal and interneuron cell groups based on the criteria described by Swadlow in the somatosensory cortex of awake rabbits [21]. It was shown that the spontaneous firing rate for interneurons was greater than two spikes per second, and the majority of interneurons had an action potential duration of less than 0.6 ms, whereas efferent neurons did not have an action potential duration of less than 0.5 ms. Since we could not be sure that we were recording the same single units across days between experiments, we analyzed single units separately for each experiment [22]. The discriminated data were processed using Neuralynx and custom software written in Matlab and Visual Basic. Peri-event histograms were constructed for each unit as well as for the experiment before and after the PTX injection. In each histogram, the baseline firing rate and the magnitude of responses were computed. The baseline firing rate was calculated for all units. A mean single-unit activity during stimulation was calculated only for units that exhibited an excitatory response. Individual normalized cell histograms (spike frequency) were pooled together for each cell type and the period of time to construct average population histograms. The stimulation-related changes were calculated within the stimulation period for single units. The sampling rate of single units was converted to 1 Hz to smooth out the deviation from the baseline.

For the PO_2_ recordings, the microwires were polarized to −0.7 V with respect to a reference electrode (located between the skull and dura), and the current was measured with a Keithley model 614 electrometer (Keithley Instruments, Cleveland, OH, USA). The output voltage from the electrometer was low pass filtered at 30 Hz, amplified, and digitized at 20 Hz. The chronically implanted PO_2_ electrodes were calibrated before implantation [23]. The sampling rate of PO_2_ was converted to a BOLD sampling rate for comparison. Neuronal activity was monitored during PO_2_ recordings.

Picrotoxin (50 µM) was dissolved in artificial cerebrospinal fluid (ACSF) for injection. This concentration of PTX was based on our preliminary studies, which demonstrated that at this concentration, there was no observable epileptiform activity on electrophysiological recordings but changes in neuronal activity are already substantial. GABA-agonist muscimol (1.75 nmol/µL) was dissolved in artificial cerebrospinal fluid (ACSF) for injection [14]. MSC injections were used to compare GABA antagonism to agonism. All injections (1 μL) were delivered through a silica tube/needle (190μm OD and 100μm ID, Polymicro Technologies, LLC, Phoenix, AZ, USA) and connected to a Hamilton syringe using transparent Tygon tubing. Equal volumes of vehicles (ACSF) were injected in the same rabbits in a randomized order (i.e., either before or after PTX injection) following the same procedure in order to control for potential injection-related effects. Single units were monitored to ensure that the volume effect was minimized [24]. If a volume effect was detected or if epileptiform activity was observed, the experiments could not proceed. BOLD and PO_2_ responses were recorded before and after (starting 15 min after the injection) pharmacological modulation. Based on our previous data [24] and data from the first group of rabbits, 15 min was sufficient to allow PTX to diffuse throughout the whisker barrel cortex.

### 2.5. Statistical Analysis

The statistical analysis of parameters derived from the BOLD, PO_2_, and neuronal responses was conducted using a two-tailed paired *t*-test. The duration of the BOLD response was defined as the full width at half maximum.

The analysis of the resting (non-stimulus related) baseline neuronal activity was conducted using a Chi-squared test, from which we compared the distribution of the neuronal activity of our treatment group (PTX injection) against our control group. The distribution consisted of three bins corresponding to an increase, decrease, or no change in the baseline firing rates. We reported decreases and increases in neuronal activity separately to avoid a situation where increased and decreased single-unit activity canceled each other out in the case of PTX injection. To identify when the PTX effect was at its maximum, we studied the dynamics of resting-state neuronal activity after PTX injections, using the consecutive five-minute intervals: 10–15, 15–20, 20–25, 25–30, and 30–35 min after injection. We used two different absolute thresholds: 30% and 10% of neuronal activity before injection to describe changes in the resting state neuronal activity after injection.

## 3. Results

### 3.1. Baseline Resting Neuronal Activity

**Figure 1:**
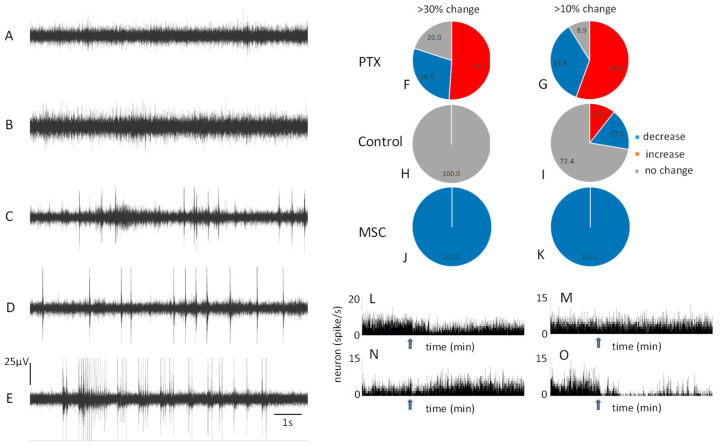
The effect of picrotoxin (PTX) on resting state neuronal activity. An example of multi-unit neuronal activity is shown before the PTX injection (**A**),after the injection of a small concentration (50 μM) of PTX (**B**), which did not cause visible resting state neuronal hypersynchronization, and larger PTX concentrations: 166 μM—(**C**), 248 μM—(**D**), and 331 μM—(**E**), which caused resting state neuronal hypersynchronization. We used the smaller 50 μM PTX concentration and two thresholds to detect changes in neuronal activity (30% and 10% changes after injection relative to the baseline before injection). After the injection of 50 μM PTX, neuronal activity experienced both an increase and decrease for both thresholds (**F**,**G**) where increases were more common. For the control experiments, changes above 30% were not observed after the injection of artificial cerebrospinal fluid (**H**) but some changes above 10% were recorded (**I**). GABA-agonist muscimol (MSC), in contrast, decreased the activity of all neurons. (**J**,**K**). Examples of peri-event histograms are shown (**L**–**O**), where PTX decreased (**L**), did not change (**M**), or increased the activity of single neurons. MSC always decreased the activity of a single neuron (**O**). Arrows indicate the time of injection.

First, we examined the dose-dependent effect of picrotoxin on neuronal activity (Figure 1). If the concentration was too high, this could have been caused by epileptic hypersynchronization [25], whereas a smaller concentration indicated less severe changes (typically increases) in the activity of neurons (Figure 1A–E).

The average firing rate from both PTX and MSC experiments before the injections was 3.21 ± 0.98 spikes/s. The analysis of the neuronal firing rate over time using a Chi-squared test revealed no statistical significance between the behavior of neurons in the 10–15, 15–20, 20–25, 25–30, and 30–35 min intervals after PTX injection (Table S1) (the number of recorded neurons was 47: an average of four units per channel). Thus, to illustrate the effect, we chose the time interval of 15–20 min after PTX injection. We used two thresholds with a 30% and 10% difference in the activity of single neurons after vs. before injection (Figure 1F,G). We observed an increase in the activity of 51% of the recorded neurons and a decrease in 29% of the neurons for the 30% threshold. We observed an increase in the activity of 56% of the recorded neurons and a decrease in 36% for the 10% threshold. Control injections produced much lower changes (Figure 1H,I). MSC decreased the neuronal activity of all single neurons (Figure 1J,K) by 71.5% on average (the number of recorded neurons was 45). Figure 1L,M illustrates examples of a single neuron’s behavior: increase (Figure 1L), no change (Figure 1M), or decrease (Figure 1N) of the firing rate after PTX injection. Figure 1O shows an example of the effect of MCS on a single neuron.

The comparison of PTX with the control injection using a chi-squared test revealed a significant difference for both effects: when the change in neuronal activity was higher/lower than 30% of the baseline (χ^2^ = 30.72, df = 2, *p* < 0.001) and 10% of the baseline (χ^2^ = 114.47, df = 2, *p* < 0.001).

### 3.2. BOLD fMRI Responses to Whisker Stimulation

**Figure 2:**
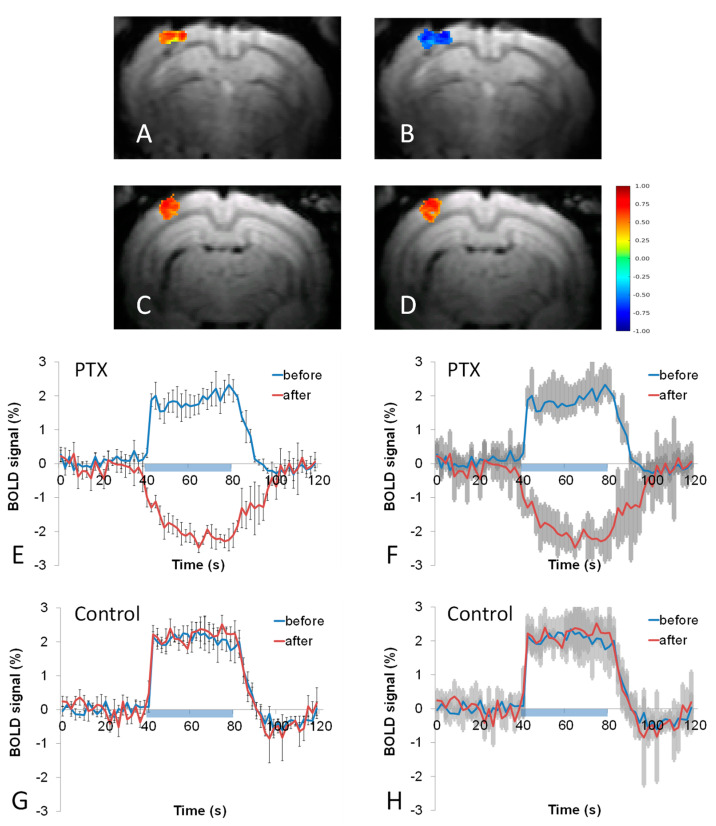
The effect of picrotoxin (PTX) on stimulus-evoked BOLD responses. Before the 50 μM PTX injection (**A**) a robust positive BOLD response was observed which extended through the depth of the cortex. After the injection of PTX, the BOLD response was negative (**B**). For control experiments with vehicle injection, the BOLD response was positive before (**C**) and after (**D**) injection. The averaged temporal profile from a region corresponding to the post-injection area exhibited a strong positive temporal profile before injection, which became negative after the injection of PTX (**E**,**F**). Both standard error bars (**E**) and 95% confidence intervals (**F**) are shown for better visibility. The average temporal profile from a region corresponding to the post-injection area exhibited a strong positive temporal profile before injection, which did not change after the control vehicle injection (**G**,**H**). Both standard error bars (**G**) and confidence intervals (**H**) are shown for the control vehicle injection. The blue bars indicate the timing of the stimulus presentation. The color bar indicates the correlation in each voxel on top of the support vector machine mask.

Neither PTX nor the control vehicle produced a significant change in the area of the BOLD response (Figure 2A,B). However, the mean BOLD response magnitude significantly decreased after PTX injection from 1.88 ± 0.28% to −1.97 ± 0.21% (*p* < 0.0003, two-tailed paired t-test) (Figure 2E,F). The control injection of the vehicle did not change the magnitude of the BOLD response (Figure 2G,H). In addition, the duration of the BOLD response did not change after PTX injections.

### 3.3. Electrophysiological Responses to Whisker Stimulation

**Figure 3:**
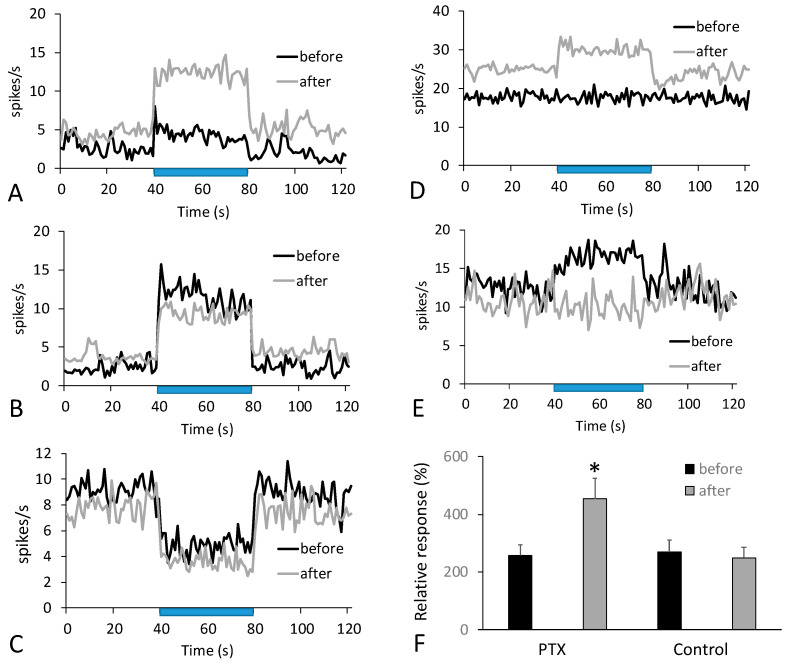
Responses of single neurons to stimulation. The typical effect of PTX was an increase in spiking activity in response to stimulation (**A**). Sometimes other behaviors were observed: a decrease in the response (**B**), or a consistent inhibitory response (**C**). Examples of a newly acquired response after the PTX injection (**D**) and abolishing the response (**E**) were associated with interneurons. The histogram (**F**) shows that the relative (to 100% baseline) magnitude of excitatory neuronal responses increased after the PTX injection but did not change after vehicle injection. The blue bars indicate the timing of the stimulus presentation. Asterisk indicates significance (*p* < 0.05).

We recorded 48 neurons responding to whisker stimulation in PTX experiments, and 71% of them exhibited solely excitatory responses. Unlike BOLD changes, we did not typically observe any change in the direction of the neuronal responses. Figure 3A illustrates the most common behavior of neurons after the injection of PTX, where the magnitude of the response increased. Figure 3B–D shows less frequent events, including inhibitory responses. The analysis of excitatory responses (Figure 3E), normalized to the baseline, used a paired *t*-test and revealed that the increase after PTX injection (by 76% compared to the response before the injection) was statistically significant (*p* < 0.02). Injections of the vehicle did not produce significant changes in neuronal responses for 46 responding neurons. Out of 48 responding neurons, 13 interneurons were found (the average action potential duration was 0.33 ± 0.02 ms, and the spiking rate was above 5 Hz). PTX had a mixed effect on the activity of these interneurons—their response either increased, was newly acquired after PTX injection (Figure 3D), or was abolished (Figure 3E).

### 3.4. PO_2_ Responses to Whisker Stimulation

**Figure 4:**
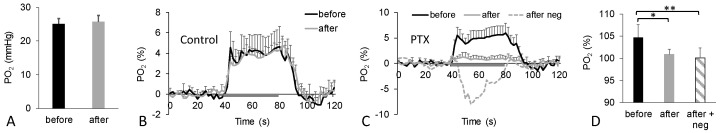
Brain tissue oxygen (PO_2_) changes after GABA-antagonist picrotoxin (PTX) injection. The baseline of PO_2_ did not change after PTX (**A**) as well as PO_2_ response to whisker stimulation after control vehicle injection (**B**). However, PO_2_ response after PTX injection was nearly abolished (grey line), and sometimes transient below-baseline PO_2_ responses were observed (**C**). An example of this transient below-baseline response is shown by the dashed line (“after neg”). The statistics for PO_2_ responses are shown on (**D**): “after” includes trials with only above-baseline responses and “after + neg” includes trials with both above- and below-baseline responses. The data on (**D**) are normalized to the 100% baseline (before stimulus). The grey bar indicates the stimulus presentation. Asterisk indicates significance (*p* < 0.05), and two asterisks indicate *p* < 0.01.

The baseline of PO_2_ did not change after PTX injection (25.12 ± 1.54 vs. 25.67 ± 1.82 mmHg before and after injection correspondently) (Figure 4A). The control injections did not change PO_2_ responses (Figure 4B). Since “negative” (below zero) PO_2_ response does not exist, we classified PO_2_ responses into two groups: above and below the baseline. PTX greatly decreased or even abolished the above-baseline PO_2_ response (104.71 ± 2.89% vs. 100.09 ± 2.22%), before and after the injection, respectively, compared to the baseline before stimulation when trials with both the above- and below-baseline responses were included (*p* < 0.009) (Figure 4C,D). The below-baseline PO_2_ response was transient and could reach an 8% decrease. It appeared only for a short period of time (typically, 1–2 trials) and did not substantially affect the average time course across 10 trials (Figure 4D).

## 4. Discussion

Our results show that picrotoxin in a low concentration generally increases neuronal activity and produces a negative BOLD response. Overall, there is a striking discrepancy between the preserved polarity of aggregate neuronal responses and changes in the polarity of the BOLD response.

The increase in neuronal activity was due to the GABA-antagonistic properties of picrotoxin, which block the output of cortical interneurons and the local network. Higher picrotoxin concentrations result in a more substantial blockade of GABA receptors, which provokes epileptiform activity due to the sensitivity of the cerebral cortex to seizures in contrast to some other structures (e.g., cerebellum) [1]. Interestingly, the GABA-agonist, MSC, consistently led to a decrease in neuronal activity, whereas the GABA-antagonist, PTX, resulted in three effects: an increase, decrease, or no change in neuronal activity, though an increase was most commonly observed (Figure 1).

The phenomenon of the negative BOLD responses to stimulation (Figure 2) has received a lot of attention due to its important implications for brain health. The neuro-vascular mechanisms underlying the negative BOLD response are not as well understood as the positive BOLD response, and its relationship to metabolic and neuronal responses is still widely debated [26]. Many studies suggest that the origin of the negative BOLD response is primarily tied to a decrease in neuronal activity below the baseline level [27,28,29,30,31,32]. In particular, the negative BOLD response has been shown to occur simultaneously with an increase in the power of the mu frequency band (EEG), which corresponds to the level of cortical inhibition [33]. Neuronal deactivation during a negative BOLD response was shown in a monkey’s visual cortex through an associated decrease in multi-unit activity [30] and similarly for the rat somatosensory cortex [32]. In humans, an association was shown between the negative BOLD response with a corresponding reduction in blood flow and decreased consumption, indicating a decrease in neuronal activity. A study investigating stimulation in the cat visual cortex demonstrated a negative BOLD response in areas neighboring the stimulated tissue, alternatively indicating that the negative BOLD response can have a vascular origin via “blood-stealing” [34]. Further studies have indicated that negative BOLD signals are driven by volume changes in CSF [35,36], along with increases in neuronal activity [37]. It has been reported that the negative BOLD response can occur in healthy humans during hand and foot movements [38]. 

Since the BOLD response depends on the dynamics of deoxyhemoglobin [39], there are two possible mechanisms for a negative BOLD response [40]. First, negative BOLD responses can occur in the presence of relatively increased oxygen consumption without concurrent functional hyperemia or, second, due to vasoconstriction, both of which affect oxygen delivery. Negative BOLD signals can accompany decreased neuronal activity as a part of the normal physiological response. However, a scenario where a negative BOLD response is observed together with increased neuronal activity (Figure 3) can be problematic for the health of the brain. The effect of picrotoxin represents a special condition when neuronal activity typically increases, but the hemodynamic response cannot properly compensate for such an increase. This condition can be seen as being close to epileptic activity due to FDIS in patients [41], and it has been suggested that unchanged or decreased BOLD responses to seizures can cause hypoxia [42]. Moreover, It is well-known that a variety of negative BOLD responses can be observed in epileptic patients [43], where FDIS is often present [41], indicating a high degree of translational value for our approach. Note that the effect of GABA antagonists is distinct from common animal models, which induce epilepsy because they use either electrical stimulation or injections of excitatory mediators (kindling) [44]. Indeed, it has been reported that kindling produces a positive BOLD response to seizures [45]. Thus, we think that the main difference between GABA antagonists and excitatory mediators lies in their effect on arterioles, where GABA antagonists can decrease vasodilation which occurs in response to stimulation.

Picrotoxin can block direct GABA connections between interneurons and arterioles [10,12,13], which would limit vasodilation, and in high concentrations of picrotoxin, these can cause profound baseline vasoconstriction in vitro [11]. Although our smaller concentration did not cause baseline vasoconstriction in vivo, which was measured by baseline PO_2_, on the other hand, negative BOLD responses could be due to the altered balance of the local inhibitory network. For example, it is known that some interneurons (e.g., somatostatin-expressing) can elicit vasoconstriction in response to a stimulus [13,46]. If their activity is enhanced while suppressing the function of interneurons which causes vasodilation (e.g., nitric oxide synthase-expressing and vasoactive intestinal peptide-expressing interneurons) and results in a negative BOLD response. This scenario is possible because the density of GABA receptors can be different in different classes of neurons, and thus, the effect of GABA antagonists on single neurons can vary. Along with this, it is possible that a vasodilation–vasoconstriction competition hypothesis, which was initially described in the cerebellum [47,48], can be applied here as well and indicates partial uncoupling between neuronal activity and the neurovascular unit [49]. Further studies involving optogenetics and measures of blood volume are needed to test these mechanisms.

Our experiments with brain tissue oxygen (Figure 4) showed that although PTX nearly abolished above-baseline PO_2_ responses to stimulation, below-baseline PO_2_ responses were transient. From a translational point of view, brain tissue oxygen is the most important measurement because it can directly indicate the level of hypoxia. Since the PO_2_ baseline did not change and the below-baseline PO_2_ response was substantial but transient, we will discuss the physiological role of stimulation-dependent excessive oxygen delivery, which was abolished after PTX injection. Initially, it was thought that stimulus-induced local blood delivery (functional hyperemia) should match increased metabolic needs in the brain tissue (for review, see [50]). However, this is not the case–the local delivery of oxygen was often excessive. It is possible that response-induced excessive oxygen delivery is needed for some specific processes. For example, the absence of functional hyperemia is associated with multiple pathologies, including ischemic stroke, Alzheimer’s disease, and hypertension [50,51]. Additionally, it was reported that a reduction in functional hyperemia by pharmacological agents resulted in a behavioral deficit in mice [52]. It was also possible that stimulation-dependent excessive oxygen delivery is a process aimed at increasing the safety of the brain. Many people live under the threat of chronic hypoxia due to, for example, heart or lung failure, pathological blood conditions, or low oxygen concentrations in the inspired air. Humans and animals constantly receive stimulation from the environment, and if the baseline of brain tissue oxygen is low, neuronal responses to stimulations can result in localized hypoxia in the absence of excessive oxygen delivery because brain tissue oxygen response can be below baseline. Thus, we can at least speak about an analog of a safety cushion when stimulation-dependent excessive oxygen delivery protects a substantial part of the population. FDIS removes this protective mechanism, and transient below-baseline oxygen responses can indicate local hypoxia when the baseline of brain tissue oxygen is low.

We can conclude that mimicking the mild functional deficiency of interneurons typically increases local neuronal activity, results in a negative BOLD response, and nearly abolishes above-baseline brain tissue oxygen responses. Further investigating the impact of this phenomenon and its underlying mechanisms will have important implications for better understanding the health of the brain.

## Data Availability

The data that support the findings of this study are available from the corresponding author upon reasonable request.

## Supplementary Materials

The following supporting information can be downloaded at: https://www.mdpi.com/article/10.3390/cells12050811/s1, Table S1 “The dynamics of the effect of picrotoxin on single neurons after the injection at 15 min”.
