# Supplementary material for: Functional Deficiency of Interneurons and Negative BOLD fMRI Response
Source: Cells. 2023 Mar 6;12(5):811. doi: 10.3390/cells12050811 (PMC10000915; doi:10.3390/cells12050811)
Supplement: Supplementary file 1 [file cells-12-00811-s001.zip › cells-2178193-supplementary.pdf]

Table S1. The dynamics of the effect of picrotoxin on single neurons after the injection at 15 min. The numbers are represented as percentage of neuron changes which are above or below the indicated 10% or 30% threshold.

	30%*			10%*		
	increase	decrease	no change	increase	decrease	no change
25-30min	44.44**	28.89	26.67	55.56	40.00	4.44
30-35min	51.11	28.89	20.00	55.56	35.55	8.89
35-40min	51.11	26.67	22.22	57.78	35.56	6.67
40-45min	46.67	31.11	22.22	55.56	37.78	6.67
45-50min	44.44	28.89	26.67	51.11	35.56	13.33

*indicates the change from the baseline before injection

** the percent of neurons which had increase, decrease or no change in the resting state activity after picrotoxin according to the corresponding threshold. Note, there is no statistical significance between time intervals.
